# Tryptase as a marker of severity of aortic valve stenosis

**DOI:** 10.1186/s12948-018-0095-6

**Published:** 2018-08-07

**Authors:** Laura M. Losappio, Corrado Mirone, Michel Chevallard, Laura Farioli, Fabrizio De Luca, Elide A. Pastorello

**Affiliations:** 1 The Department of Allergology and Immunology, A.S.S.T. Grande Ospedale Metropolitano Niguarda, Milan, Italy; 2 The Department of Laboratory Medicine, A.S.S.T. Grande Ospedale Metropolitano Niguarda, Milan, Italy; 3 Unit of Allergy and Immunology, Niguarda Ca’ Granda Hospital, Piazza Ospedale Maggiore, 3, 20162 Milan, Italy

**Keywords:** Aortic valve stenosis, Biomarker, Tryptase

## Abstract

**Background:**

Severe aortic valve stenosis is one of the most common cause of mortality in adult patients affected with metabolic syndrome, a condition associated with an active inflammatory process involving also mast cells and their mediators, in particular tryptase. The aim of this study was to characterize the possible long-term prognostic role of tryptase in severe aortic valve stenosis.

**Case presentation:**

The baseline serum tryptase was measured in 5 consecutive patients admitted to our Hospital to undergo aortic valve replacement for severe acquired stenosis. Within 2 years after, the patients were evaluated for the occurrence of major cardiovascular events (MACE). The tryptase measurements were higher in patients experiencing MACE (10.9, 11.7 and 9.32 ng/ml) than in non-MACE ones (5.69 and 5.58 ng/ml).

**Conclusions:**

In patients affected with severe aortic stenosis, baseline serum tryptase may predict occurence of MACE. Further studies are needed to demonstrate the long-term prognostic role of this biomarker.

## Background

Severe aortic valve stenosis is one of the most common cause of mortality in adult patients affected with metabolic syndrome [1], i.e. a clinical condition characterized from visceral obesity that is traduced into insulin resistance, atherogenic dyslipidemia and proinflammatory state [2]. It is frequently due to an active process involving several pathways, including lipid infiltration, chronic inflammation, fibrosis formation, osteoblasts activation, and valve mineralization. Other causes are congenital valve defects, systemic inflammatory diseases, and endocarditis [3]. Prevalence is between 2 and 9% in subjects over 65 years and it increases significantly in forthcoming decades as a consequence of the ageing population and of more accurate diagnostic methods [4]. Severe aortic stenosis is defined by the presence of maximum aortic velocity ≥ 4 m/s, or aortic valve area ≤ 1.0 cm^2^, or by the presence of severe leaflet calcification and severely reduced leaflet opening. Surgical aortic valve replacement is indicated in symptomatic patients with severe high-gradient aortic stenosis, and in asymptomatic ones with severe aortic stenosis and left ventricular ejection fraction < 50% [5]. Its natural history results in the obstruction of the left ventricular outflow, followed by pressure overload and compensatory hypertrophy of the left ventricle. Excessive hypertrophy may decrease coronary blood flow reserve, increase collagen synthesis, interstitial fibrosis, and myocyte degeneration resulting in ischemic cardiac disease, sudden death and heart failure syndrome. Moreover, these patients have major risk of bleeding due to angiodysplasia, alterated platelets function and low concentration of von Willebrand factor [3]. High-sensitivity cardiac troponin T (hsTnT) is usefulness for risk stratification of severity and mortality [6]. However, recently some authors described the role of mast cells in calcified aortic stenosis [7] and an autoptic study detected these cells in the excised valves of patient undergoing elective aortic valve replacement in comparison with normal aortic valves from five healthy subjects obtained on autopsy served as negative controls [8]. In light of the above, we studied basal serum tryptase as a new serological prognostic biomarker in aortic valve stenosis. Tryptase is a mast cell serine protease that provides information about mast cell number, distribution, and activation depending on the clinical context [9]. In some cardiovascular diseases, this enzyme has important implications and represents an index of mast cells’ burden [10, 11]. In particular, in subjects affected with acute coronary syndrome we found higher basal tryptase values in so defined ‘cardiovascular complex’ patients than in ‘non-complex’ ones [12]. Moreover, in the same population the basal serum tryptase was significantly correlated to the development of major cardiovascular events’ (MACE) up to 2 years, demonstrating a possible long-term prognostic role of this biomarker [13].

## Cases report

Herein, we described a total of 5 consecutive patients admitted to our Hospital from January 2015 to December 2016, to undergo aortic valve replacement for severe acquired stenosis. None was affected with autoimmunity diseases, severe allergies, cancer, renal failure, mastocytosis, refractory anemia, myelodysplastic syndromes, and hypereosinophilic syndrome. After admission, we collected from all the patients medical history, echo-cardiogram, serum tryptase, C-reactive protein, hsTnT, plasma glucose, and lipid parameters. Serum tryptase levels were measured by ImmunoCAP tryptase in vitro fluoro-enzyme-immunoassay test (Phadia, now Thermo Fisher Scientific, Uppsala, Sweden), according to the manufacturer’s instruction. Within 2 years after the aortic valve replacement, the patients were evaluated for the occurrence of MACE including myocardial infarction, cardiac arrhythmias, stroke, systemic embolism, heart failure and sudden death. Table 1 shows patients’ clinical characteristics. At 2-year follow up, 3 patients experienced MACE: 1 died and 2 had acute coronary syndrome. In these patients tryptase levels were 10.9, 11.7 and 9.32 ng/ml respectively, about twofold higher than in non-MACE ones: 5.69 and 5.58 ng/ml.

**Table 1:** Clinical characteristics of study patients

Sex/age, years	MACE-patients			Non-MACE patients	
	M/85	F/58	F/53	F/72	F/77
Clinical history					
Hypertension	Yes	Yes	Yes	Yes	Yes
Hypercolesterolemia	No	Yes	No	Yes	Yes
Currently smoking	Yes	No	No	No	No
Diabetes mellitus	No	Yes	No	No	No
Obesity	No	No	No	No	Yes
COPD	Yes	No	No	No	No
Left ventricle ejection fraction during index hospitalization					
Ejection fraction (%)	30	58	56	40	60
Mean transvalvular gradient (mmHg)	70	75	70	65	65
Diagnostic findings					
Tryptase, ng/ml	10.9	11.7	9.32	5.69	5.58
CRP, mg/dl	3.2	0.8	0.2	0.3	1.1
hsTnT, ng/l	49.5	8.0	7.9	40	7.2
Serum triglycerides, mg/dl	117	189	150	176	68
HDL cholesterol, mg/dl	32	40	38	37	72
LDL cholesterol, mg/dl	90	97	100	116	89
Plasma glucose, mg/dl	119	181	104	87	114
Major cardiovascular events	Sudden death	STEACS	STEACS	None	None

*STEACS* ST elevation acute coronary syndrome, *COPD* Chronic obstructive pulmonary disease, *CRP* C-reactive protein, *hsTnT* high-sensitivity cardiac troponin T, *HDL* high-density lipoprotein, *LDL* low-density lipoprotein, *MACE* major cardiovascular events

## Conclusions

Our results could be in agreement with the literature of the last few decades, in which a relationship between high tryptase levels and the development of MACE in acute coronary syndrome patients was found, to demonstrate the tryptase role as a marker of the inflammatory and atherosclerotic process [13, 14]. Indeed, in stenotic aortic valves mast cells secrete tryptase, chymase, cathepsin G and vascular endothelial growth factor inducing extracellular matrix degradation and valvular neovascularization [15].

In conclusion, we hypothesized that high tryptase levels may be a risk factor of development of MACE in severe aortic stenosis. Further studies on largest populations are required to confirm this hypothesis.

## Abbreviations

MACE: major cardiovascular events
hsTnT: high-sensitivity cardiac troponin T

## References

[1] Pagé A, Dumesnil JG, Clavel MA, Chan KL, Teo KK, Tam JW, Mathieu P, Després JP, Pibarot P, ASTRONOMER Investigators (2010). Metabolic syndrome is associated with more pronounced impairment of left ventricle geometry and function in patients with calcific aortic stenosis: a substudy of the ASTRONOMER (Aortic Stenosis Progression Observation Measuring Effects of Rosuvastatin). J Am Coll Cardiol..

[2] Després JP, Lemieux I, Bergeron J, Pibarot P, Mathieu P, Larose E, Rodés-Cabau J, Bertrand OF, Poirier P (2008). Abdominal obesity and the metabolic syndrome: contribution to global cardiometabolic risk. Arterioscler Thromb Vasc Biol.

[3] Olszowska M (2011). Pathogenesis and pathophysiology of aortic valve stenosis in adults. Pol Arch Med Wewn.

[4] Townsend CM (2008). Sabiston textbook of surgery.

[5] Nishimura RA, Otto CM, Bonow RO, Carabello BA, Erwin JP, Guyton RA, O’Gara PT, Ruiz CE, Skubas NJ, Sorajja P, Sundt TM, Thomas JD, American College of Cardiology/American Heart Association Task Force on Practice Guidelines (2014). 2014 AHA/ACC guideline for the management of patients with valvular heart disease: executive summary: a report of the American College of Cardiology/American Heart Association Task Force on Practice Guidelines. J Am Coll Cardiol.

[6] Dahou A, Clavel MA, Capoulade R, O’Connor K, Ribeiro HB, Côté N, Le Ven F, Rodés-Cabau J, Dumesnil JG, Mathieu P, Pibarot P (2017). B-type natriuretic peptide and high-sensitivity cardiac troponin for risk stratification in low-flow, low-gradient aortic stenosis: a substudy of the TOPAS study. JACC Cardiovasc Imaging..

[7] Steiner I, Krbal L, Rozkoš T, Harrer J, Laco J (2012). Calcific aortic valve stenosis: immunohistochemical analysis of inflammatory infiltrate. Pathol Res Pract.

[8] Wypasek E, Natorska J, Grudzień G, Filip G, Sadowski J, Undas A (2013). Mast cells in human stenotic aortic valves are associated with the severity of stenosis. Inflammation..

[9] Valent P (2013). Mast cell activation syndromes: definition and classification. Allergy.

[10] Searle J, Danne O, Müller C, Mockel M (2011). Biomarkers in acute coronary syndrome and percutaneous coronary intervention. Minerva Cardioangiol.

[11] Kounis NG, Tsigkas G, Almpanis G, Kounis GN, Mazarakis A, Hahalis G (2011). Tryptase levels in coronary syndrome and in hypersensitivity episodes: a common path was towards Kounis syndrome. Atherosclerosis..

[12] Morici N, Farioli L, Losappio LM, Colombo G, Nichelatti M, Preziosi D, Micarelli G, Oliva F, Giannattasio C, Klugmann S, Pastorello EA (2016). Mast cells and acute coronary syndromes: relationship between serum tryptase clinical outcomes and severity of coronary artery disease. Open Heart..

[13] Pastorello EA, Farioli L, Losappio LM, Morici N, Di Biase M, Nichelatti M, Schroeder JW, Balossi L, Klugmann S (2015). Serum tryptase detected during acute coronary syndrome is significantly related to the development of major adverse cardiovascular events after 2 years. Clin Mol Allergy..

[14] Helske S, Lindstedt KA, Laine M, Mäyränpää M, Werkkala K, Lommi J, Turto H, Kupari M, Kovanen PT (2004). Induction of local Angiotensin II producing systems in stenotic aortic valves. J Am Coll Cardiol.

[15] Syva¨ranta S, Helske S, Laine M, Lappalainen J, Kupari M, Ma¨yra¨npa¨a¨ MI, Lindstedt KA, Kovanen PT (2010). Vascular endothelial growth factor-secreting mast cells and myofibroblasts: a novel self-perpetuating angiogenic pathway in aortic valve stenosis. Arterioscler Thromb Vasc Biol.

